# Partial intracapsular tonsillectomy in the treatment of pediatric obstructive sleep apnea/hypopnea syndrome: a prospective study with 5-year follow-up

**DOI:** 10.1007/s00405-021-07119-3

**Published:** 2021-10-10

**Authors:** Matteo Cavaliere, Pietro De Luca, Egidio De Bonis, Riccardo Maurizi, Claudia Cassandro, Massimo Ralli, Ettore Cassandro, Alfonso Scarpa

**Affiliations:** 1 Department of Medicine, Surgery and Dentistry, University of Salerno, Salerno, Italy; 2 Department of Clinical Sciences and Translational Medicine, Otorhinolaryngology Unit, Tor Vergata University of Rome, Rome, Italy; 3 Department of Surgical Sciences, University of Turin, Turin, Italy; 4 Department of Surgical Sciences, Sapienza University of Rome, Rome, Italy

**Keywords:** Tonsillotomy, Bipolar forceps, Tonsillectomy, OSAHS, Paediatric, OSA-18, Quality life, Snoring

## Abstract

**Objective:**

To assess efficacy and safety of tonsil reduction with bipolar forceps electrocautery as treatment of paediatric obstructive sleep apnea/hypopnea syndrome (OSAHS).

**Study design:**

Prospective interventional study.

**Methods:**

Two hundred and sixty-three children aged 4–10 years with OSAHS and an apnea hypopnea index (AHI) > 3 were enrolled from March 2013 to January 2016. Pre-operative evaluation included oropharyngeal clinical examination with fiberoptic nasopharyngoscopy, OSA-18 questionnaire and overnight sleep study. All children were treated with adenoidectomy and tonsillotomy with bipolar forceps. OSA-18 questionnaire and overnight sleep study were performed 30 days after surgery.

**Results:**

Pre-operative average of the OSA-18 questionnaires was of 70.3 (SD = 9.7); 30-day post-operative score was 23.15 (SD = 8.2; *p* = 0.045). Pre-operative average Apnea Hypopnea Index (AHI) score was 9.41 (SD = 4.1); 30-day post-operative average of AHI score was of 1.75 (SD = 0.8; *p* = 0.012). Oxygen Desaturation Index (ODI) rate changed from 7.39 (SD = 4) to 1.34 (30-day post-operative) (SD = 4.7; *p* = 0.085). NADIR rate changed from 79% (SD = 6.32) to 90% (30-day post-operative) (SD = 5.18; *p* = 0.00012). Peri- and post-operative complications in our sample were mainly pain (average 75 doses of paracetamol), while bleeding did not occur (0%). All patients received a follow-up examination 5 years after surgery to evaluate tonsil size; at this time-point, a reduction in tonsil size from 3.6 (3–4; SD = 4.2) to 1.3 (1–2; SD = 5.5) was found, while tonsil regrowth was observed in five children (2%).

**Conclusion:**

This study showed that partial tonsillotomy with bipolar forceps electrocautery associated to adenoidectomy is an effective technique in treating OSAHS symptoms in children and ensures less complications in terms of hemorrhage, postoperative pain and infections compared to traditional adenotonsillectomy. The very low tonsillar regrowth rate reported in this study may support the routine use of this technique.

## Introduction

Paediatric obstructive sleep apnea/hypopnea syndrome (OSAHS) in defined as a disorder of breathing during sleep characterized by prolonged partial upper airway obstruction and/or intermittent complete obstruction that affects regular ventilation during sleep and normal sleep patterns [1]. OSAHS prevalence is 1.2–5.7% among paediatric population and reaches 36% in obese children [2]. Main risk factors for OSAHS in paediatric population are adeno-tonsillar hypertrophy, obesity, craniofacial malformations, Down Syndrome and neuromuscular diseases [3]. Complications range from cardiovascular problems [4, 5] to developmental delays, with clear prevalence of the latter [6].

Corticosteroids (alone or in combination), anti-leukotrienes, and continuous positive airway pressure (CPAP) have been widely evaluated during decades as OSAHS therapy, but only surgery seems to resolve symptoms linked to adenotonsillar hypertrophy [7–10].

For many years, tonsillectomy with/without adenoidectomy has been the most common surgical procedure performed in children with OSAHS due to tonsillar and/or adenotonsillar hypertrophy, representing about 15% of surgical procedures performed in paediatric population under the age of 15. The main complication of tonsils surgery is post-tonsillectomy bleeding [11–13].

Recently, there has been a rising interest in partial, sub-total, and intra-capsular tonsillectomy—also known as tonsillotomy—a procedure that can be performed with many surgical instruments as microdebrider, CO_2_ laser, coblator, and monopolar or bipolar forceps [14]. This technique has been reported in both adults and in children [15–21].

The aim of this prospective study was to assess efficacy and safety of tonsil reduction with bipolar forceps electrocautery as treatment of paediatric OSAHS with a 5-year follow-up.

## Materials and methods

A prospective study on children suffering from OSAHS was performed. The study protocol was approved by ethics committee of Campania Centro and informed written consent was obtained from the patients’ parents.

### Patients

From March 2013 to January 2016, 345 children (age range 4–10 years) referred to our Otolaryngology Unit for uncomplicated childhood OSAHS associated with adeno-tonsillar hypertrophy were consecutively enrolled in this study. Children with a body mass index (BMI) > 25, craniofacial abnormalities, heart or lung pathology or recurrent throat infections were excluded from the study. Eighty-two patients were not included in the study because of missing follow-up; 263 patients completed follow-up and were included in the study.

### Clinical and follow-up procedures

At admission, clinical data including age, sex, weight and BMI were recorded. Physical examination was performed by three different clinicians with fiberoptic nasopharyngoscopy; the tonsil size was evaluated before and at the end of the follow-up period (5 years) after surgery using the Brodsky scheme (from grade 1 to grade 4) [17].

OSA-18 questionnaire [18] was administered to parents before surgery and 30 days after surgery. It assessed sleep disorders, including snoring (4 questions), physical suffering (4 questions), emotional distress (3 questions), daytime problems (3 questions) and caregiver concerns (4 questions); we asked parents to assign, for each point, a score from 1 to 7 for a maximum score of 126.

Post-operative pain was self-evaluated by children using a visual analogue scale (VAS) ranging from zero (no pain) to 10 (the worst possible pain) during the first 30 days after surgery. Paracetamol was administered for pain; parents were asked to report the analgesic dose taken every day.

Parents were asked to subjectively evaluate child’s snoring during the first 30 nights after surgery using a VAS score ranging from zero (no snoring) to 10 (the worst possible snoring).

Outpatient sleep study with ApneaLink Air (ResMed, Germany) was performed in all patients before and 30 days after surgery. It consisted in a nasal cannula detecting flow and a pulse oximeter for reveal O_2_ saturation during sleep and heart rate. Parameters assessed were Apnea Hypopnea Index (AHI) corresponding to average of apneas and hypopneas per hour of sleep; Oxygen Desaturation Index (ODI) which was expression of the number of desaturation events (> 3%) per hour of sleep; and O_2_ NADIR (the lower O_2_ saturation level). We considered eligible for surgery only patients with AHI > 3 [19].

### Surgical technique

Surgery was performed under general anesthesia with endotracheal intubation; patients were in the Rosen position for an optimal exposure of the lower pole of both tonsils. Adenoidectomy was firstly performed, followed by tonsillotomy. Possible caseous or necrotic material was removed by Yankauer aspirator, and the tonsils were cauterized using a high frequency electric generator (Autocon® III 400, Karl Storz, Germany) with the cauterization power at 15 W. The active part of bipolar forceps with angled tips (1 cm long and 2 cm thick), spread to 1 cm was introduced inside the tonsillar parenchyma, and a variable number (from 5 to 15, depending on tonsillar dimension) passages were performed until tissue necrosis (generally it takes 5 s per passage to obtain necrosis). Cauterization was considered ended when tonsils were reduced by about a third of initial dimension, paying a lot of attention to avoid damages of pillars and pharynx. Surgery was performed by a single surgeon.

### Statistical analysis

MedCalc Statistical Software version 14.8.1 (MedCalc Software Ltd, Ostend, Belgium; http://www.medcalc.org; 2014) was used to perform statistical analysis. The *t* test was used to compare the numerical values at different timepoints. A *p* value < 0.05 was considered for statistical significance.

## Results

Two hundred sixty-three patients completed the 5-year follow-up, while 82 patients did not complete the follow-up period and were not included. For children included in the study, the mean age was 6.74 (4–10 years; SD = 1.9); 141 subjects (53.6%) were males, while 122 (46.4%) were females. Average weight was 29.6 kg (13.3–50.4 kg; SD = 6.3). The mean BMI was 21.4 (18–25; SD = 8). For children that were not included due to missing follow-up, the mean age was 7.11 (4–11 years; SD = 3.1); 46 subjects (56.8%) were males and 35 (43.2%) were females. Average weight was 27.2 kg (12.9–47.9 kg; SD = 8.9). The mean BMI was 19.8 (17–24; SD = 9.4). There were no significant differences for age, sex, weight and BMI between patients included in the study and patients excluded for missed follow-up (*p* > 0.05 for each item).

Tonsil size was evaluated before and 5 years after surgery; at the 5-year follow-up a reduction in tonsil size from 3.6 (3–4; SD = 4.2) to 1.3 (1–2; SD = 5.5) was found, while tonsil regrowth was observed in five children (2%).

Results of the OSA-18 questionnaire showed an average pre-operative score was 70.36 (49–104; SD = 9.7); 30-day post-operative score was 23.15 (19–37; SD = 8.2). The difference was statistically significant (*p* = 0.0425).

Results from sleep study are shown in Table 1. Pre-operative AHI mean value was 9.41 (SD = 4.1), while 30-day post-operative AHI mean value was 1.75 (SD = 0.8); this result was statistically significant (*p* = 0.012). O_2_ NADIR rate changed from 79%, SD = 6.32 (pre-operative) to 90%, SD = 5.18 (30-day post-operative) (*p* = 0.00012); ODI rate changed from 7.39, SD = 4 (pre-operative) to 1.34, SD = 4.7 (30-day post-operative) (*p* = 0.085).

**Table 1:** Results of the sleep study with polygraphy pre-operatively and 30 days after surgery

	Pre-operative (*n* = 263)	Post-operative (*n* = 263)	*p* value
Apne Hypopnea Index (AHI)			
Mean	9.41	1.75	0.012
Median	6.7	1.5	
Range	3.9–42.1	0.3–4.5	
SD	4.1	0.8	
Oxygen Desaturation Index (ODI)			
Mean	7.39	1.34	0.085
Median	6.2	1	
Range	2–43.9	0.5–3.8	
SD	4	4.7	
Oxygen NADIR			
Mean	79%	90%	0.00012
Median	80%	92%	
Range	53–91%	67–95%	
SD	6.32	5.18	

Parents’ subjective evaluation of child’s snoring during the first 30 nights after surgery revealed that snoring was more common in the first 3–5 days after the surgery due to post-surgical edema and it gradually declined.

VAS scale reported from parents showed an average of 75 doses of paracetamol taken by our patients during the first post-operative month, with a progressive decrease over time. Two children (6%) developed upper respiratory tract infections (one otitis media, one acute sinusitis).

## Discussion

The comparison of OSA-18 questionnaire and overnight sleep study results pre- and 30-day post-operatively showed that tonsillotomy technique using bipolar forceps may be an effective technique to improve sleep parameters and the overall OSAHS symptomatology in children between 4 and 10 years old. Shaul and Attal [20] performed tonsillotomy in 30 patients (2 to 15 years old) and assessed a postoperative evaluation using the “PSQ score” questionnaire and sleep study showing encouraging results; our study confirmed the results from the work of Shaul and Attal, but enrolled a significantly larger number of patients, with a longer follow-up time. According to the meta-analysis from Wang et al. [21] and to the systematic review conducted by Zhang et al. [22], a long follow-up is strictly required to draw conclusions about the real efficacy of partial tonsillotomy.

The evidence from the study of Mukhatiyar et al. [23] are similar to our results; the authors showed an AHI reduction from 17 to 1.7. Our findings are also supported by the result of Choi et al. [24], that showed an AHI reduction from 12.5 to 1.9 in children undergoing tonsillotomy. Both studies suggested a complete resolution of the symptomatology after partial tonsillotomy.

Compared to traditional tonsillectomy, tonsillotomy seemed to ensure a better quality of life in terms of postoperative pain after the first postoperative week, with patients requiring less analgesic to control post-operative pain. Grab and Harhash [25] reported a significant pain reduction 10 days after surgery, an average of 3 days more compared to tonsillotomy.

Our patients took an average of 75 doses of paracetamol during the first 30 days after surgery; Hultcrantz et al. [26] reported a total amount of 16 doses of paracetamol and Vicini and Eesa [27] reported a total dose of 15.7; however, the size of the cohorts is not comparable.

In their study, Ericcson et al. [28] reported that 33% of treated patients had at least one episode of fever in the 6 months after surgery; after tonsillotomy, the upper airway infections rate seemed to be lower than after total tonsil removal. In our study, only 6% of the patients suffered from an upper respiratory tract infection during the first post-operative year.

No intra-operative bleeding was reported during surgical procedures; this may be due to the reduced caliber of vessels damaged by intracapsular resection compared to tonsillectomy. In addition, these vessels were immediately coagulated during the reduction procedure. No post-operative bleeding was noted in our sample. Previously, other authors conducted studies on post-tonsillotomy bleeding; Vicini et al. [27] performed tonsillectomies with microdebrider and reported 3 cases of secondary bleeding on 251 children; Morinier et al. [29] performed 88 tonsillectomies with radiofrequencies and noticed 3 episodes of bleeding. As reported in several studies, the hemorrhage rate may vary according to the technique used [30, 31].

Literature reported a tonsil regrowth in a percentage ranging from 3.33% (confidence interval 95% = 1.62–6.82, *p* = 0.001) to 6% [30, 31]. Eviatar et al. [32] performed a 10-year follow study on 39 children undergoing partial or total tonsillectomy and reported a 5% of recovery rate after partial tonsillotomy and 0% after total tonsillectomy. We observed a tonsil regrowth in five children (2%).

Economic analysis of the operative costs shows a cost of 420€ and 21-min surgery time for each adenoidectomy and tonsillotomy and a cost of 552 € and an average of 27.6-min surgery time for each adenotonsillectomy. The cost for the microdebrider blade is between 70 and 90 € while the cost for coblation ranges between 120 and 180 €. The cost for bipolar forceps is nearly 400€ but can be reprocessed and re-used up to 500 times. Therefore, the coblation procedure is nearly 75% more expensive than a tonsillotomy with bipolar forceps [33–35].

### Limits and strengths of the study

The main limitation of this study is the absence of a control group of patients treated with traditional tonsillectomy. In addition, the instrument used for sleep parameter evaluation (ApneaLink Air) is a screening tool that has a lower sensitivity compared to professional polysomnography technologies. Last, the current study design cannot rule out that recorded benefits were attributable to adenoidectomy only, without a specific contribution of tonsillotomy. The strengths include the prospective nature of the study, the number of children treated, and the 5-year follow-up for all patients for tonsil size and regrowth.

## Conclusions

This study showed that partial tonsillotomy with bipolar forceps electrocautery is an effective technique in treating OSAHS symptoms in paediatric population with less complications in terms of hemorrhage, postoperative pain, and infections during the first year after surgery. Moreover, the tonsillar regrowth rate at 5-year follow-up reported in this study may justify the routine use of this technique. Postoperative risk of tonsils regrowth should be clearly discussed with patients and their parents before proceeding with partial tonsillotomy.
